# Real Time Ligand-Induced Motion Mappings of AChBP and nAChR Using X-ray Single Molecule Tracking

**DOI:** 10.1038/srep06384

**Published:** 2014-09-16

**Authors:** Hiroshi Sekiguchi, Yasuhito Suzuki, Yuri Nishino, Suzuko Kobayashi, Yoshiko Shimoyama, Weiyan Cai, Kenji Nagata, Masato Okada, Kouhei Ichiyanagi, Noboru Ohta, Naoto Yagi, Atsuo Miyazawa, Tai Kubo, Yuji C. Sasaki

**Affiliations:** 1 CREST Sasaki Team, Japan Science and Technology Agency, The University of Tokyo, #609 Kiban Bldg., 5-1-5 Kashiwanoha, Kashiwa City, Chiba, 277-8561, Japan; 2 Research & Utilization Division, Japan Synchrotron Radiation Research Institute, SPring-8 1-1-1 Kouto, Sayo-cho, Sayo-gun, Hyogo 679-5198, Japan; 3 Graduate School of Frontier Sciences, The University of Tokyo, Kiban Bldg., 5-1-5 Kashiwanoha, Kashiwa City, Chiba, 277-8561, Japan; 4 Graduate School of Life Sciences, University of Hyogo, 3-2-1 Kouto, Kamigori-cho, Ako-gun, Hyogo, 679-1297, Japan; 5 RIKEN SPring-8 Center, 1-1-1 Kouto, Sayo-cho, Sayo-gun, Hyogo, 679-5148, Japan; 6 Biomedical Research Institute, National Institute of Advanced Industrial Science and Technology (AIST), 1-1-1 Higashi, Tsukuba, Ibaraki 305-8566, Japan; 7 Molecular Profiling Research Center for Drug Discovery, National Institute of Advanced Industrial Science and Technology (AIST), 2-4-7 Aomi, Koto-ku, Tokyo 135-0064, Japan

## Abstract

We observed the dynamic three-dimensional (3D) single molecule behaviour of acetylcholine-binding protein (AChBP) and nicotinic acetylcholine receptor (nAChR) using a single molecule tracking technique, diffracted X-ray tracking (DXT) with atomic scale and 100 μs time resolution. We found that the combined tilting and twisting motions of the proteins were enhanced upon acetylcholine (ACh) binding. We present the internal motion maps of AChBP and nAChR in the presence of either ACh or α-bungarotoxin (αBtx), with views from two rotational axes. Our findings indicate that specific motion patterns represented as biaxial angular motion maps are associated with channel function in real time and on an atomic scale.

In addition to the static crystallographic information regarding a protein's structure, dynamic information regarding a protein's conformational changes would be helpful in elucidating the molecular mechanisms that regulate protein functions, such as ion channel gating and ligand-induced receptor activation. Such dynamic information can be obtained using recently developed single molecule techniques^1^ and can be predicted using molecular dynamics simulations^2^. To improve the monitoring precision and signal-to-noise ratio under physiological conditions, we have proposed a single molecule technique that utilises short wavelength probes, such as X-rays^3^,^4^ and electrons^5^, to monitor the small internal motions of a single protein. This technique, diffracted X-ray tracking (DXT)^6^, was used to successfully monitor the dynamic twisting motion of KcsA, a pH-sensitive potassium channel, upon gating^6^. In DXT, the internal motion of an individual single protein is monitored through the trajectory of a Laue diffraction spot from a nanocrystal attached to the immobilised target protein (Figures 1a and 1b). The remarkable feature of DXT is its ability to monitor the rotational motion of the nanocrystal at several milliradian scales with two rotational axis views, tilting (θ) and twisting (χ) motions (Figure 1c)^7^,^8^.

Here, using DXT with improved time resolution, we performed real-time single molecule observations of muscle-type nAChR, one of the most thoroughly studied members of the pentameric ligand-gated ion channel (pLGIC) family^9^,^10^, and AChBP, a homologue to the extracellular ligand-binding domain of nAChR that is often used as a model for the ligand-binding domain of pLGICs^11^,^12^. Based on electron crystallographic structural information, it was expected that the conformational changes of nAChR associated with channel opening would cause twisting and tilting motions of both the ligand-binding and channel domains^13^,^14^. In prokaryotic pLGICs, it has been suggested based on X-ray crystallography that channel opening involves a tilting motion of the channel domain^15^,^16^,^17^. Because DXT can obtain information on phenomena such as the tilting (θ) and twisting (χ) motions of target proteins, as shown in Figure 1c, we applied this technique to investigate the gating mechanism of nAChR. In this article, we focused on ligand-induced motions of nAChR, using an immobilised gold nanocrystal on the ligand-binding domain of nAChR and on nAChR's extracellular homologue, AChBP.

## Results

**Figure 1:**
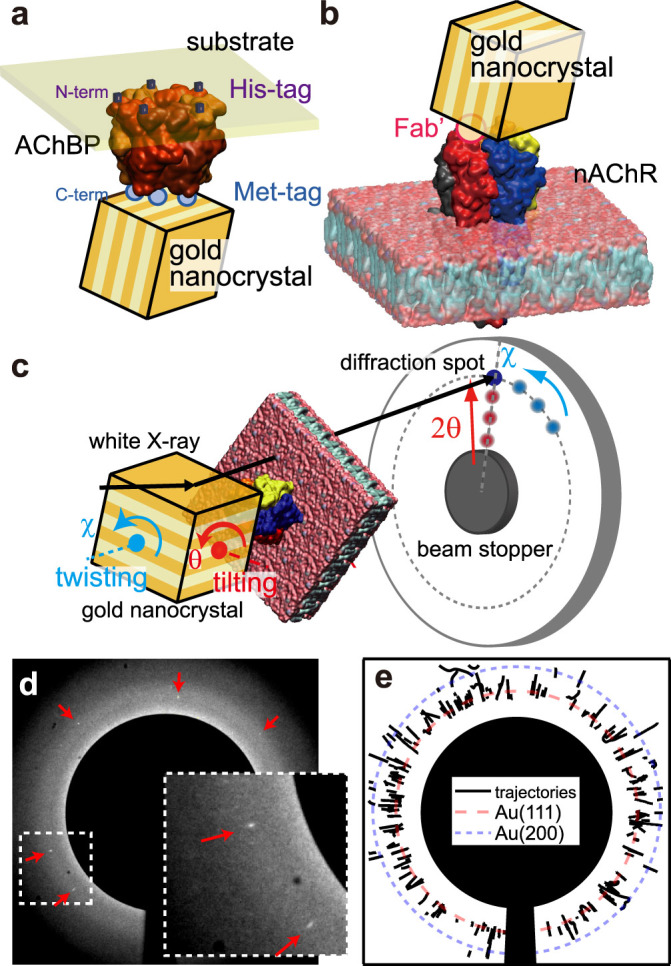
DXT measurements of AChBP and nAChR. (a–b): Schematic of the immobilisation of gold nanocrystals on AChBP (a) and nAChR (b). (c): Schematic of the tilting (θ) and twisting (χ) motions observed by DXT. Actual DXT diffraction image on AChBP with gold nanocrystal (d) and trajectories of diffraction spots in DXT experiment in the presence of ACh (e). Diffraction spots indicated with red arrows (d) were tracked, and their trajectories (e, black solid lines) appeared surrounding the expected positions of Au (111) (dashed red line) and Au (200)(dotted blue line) based on the major incident X-ray energy (15.2 keV) and sample-to-camera distance (100 mm). The insert image at the lower right in (d) is an enlargement of the dashed square located at the lower left in (d).

We observed the dynamic 3D single molecule behaviour of AChBP and nAChR using a single molecule tracking technique, DXT. Recombinant AChBP and native nAChR, which was isolated from the membrane of the *Torpedo californica* electric organ, were immobilised on substrate surfaces using His-tag chemistry and a bifunctional crosslinker, respectively. The quaternary structure of both proteins was maintained through the sample preparation and measurement process. To monitor the internal motion of the proteins from multiple orientations, we controlled the position of the nanocrystal labels on the two protein molecules. The nanocrystal was immobilised on the C-terminus of AChBP via a Met-tag (Figure 1a) and on the F(ab')2 fragment of the monoclonal antibody (mAb) 35, which recognises the extracellular side of the nAChR α-subunit^18^ (Figure 1b). The antibody used has been shown to have no adverse effect on the ligand binding or channel gating of nAChR.

**Figure 2:**
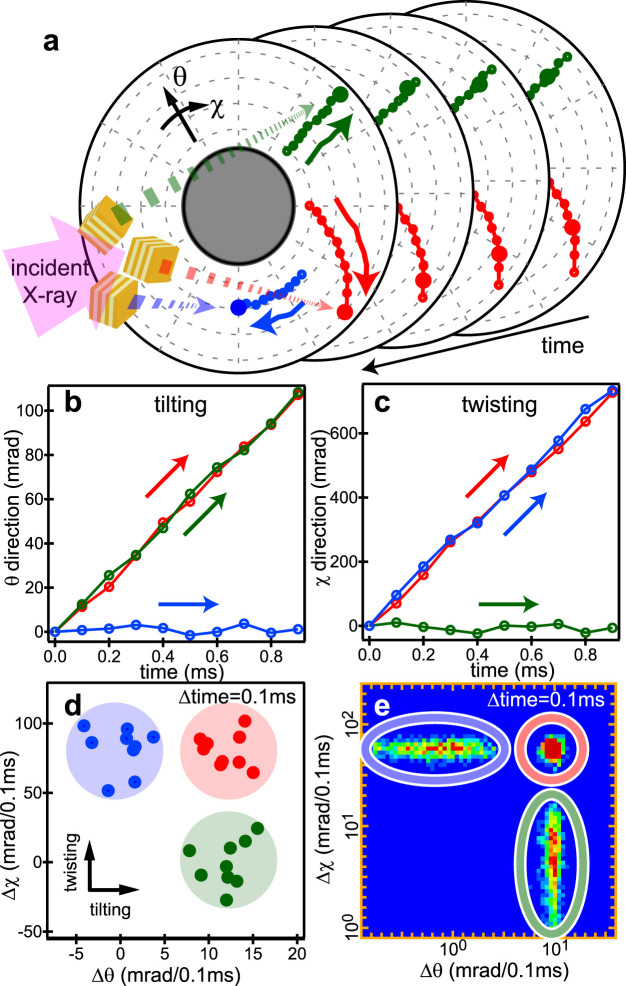
DXT analysis procedures from diffraction spot trajectory to a 2D-internal motion map. (a): Schematics of the diffraction spot trajectories in time-resolved diffraction images. Three examples of diffraction spot trajectories from gold nanocrystals exhibiting simple twisting motion (blue), simple tilting motion (green), and both tilting and twisting motions (red) are shown. (b–c): The displacement of angular position as a function of time in the tilting (b) and twisting (c) directions. (d): 2D scatterplot of the protein's internal motion generated from angular displacement in both the tilting and twisting directions in fixed time interval, e.g., 100 μs. (e): 2D-internal motion map of the protein. Logarithm of absolute displacement within a certain time interval was used to draw the 2D-histogram, which was normalised to obtain the 2D-internal motion map (probability density map).

We analysed the angular displacement per unit time in two axes, tilting (θ) and twisting (χ), as shown in Figure 1c, and compared the motion of the nanocrystal on the target protein under three different experimental conditions: in the presence of acetylcholine (ACh, 100 μM), in the presence of α-bungarotoxin (αBtx, 200 nM), and in the absence of any ligand (free). The DXT measurement was performed at BL40XU in SPring-8 Japan with the optics shown in Supplementary Figure S1 and a time resolution of 100 μs. The size of the incident X-ray beam at sample position was 40 μm (vertical) and 150 μm (horizontal) in full width at half maximum, and all gold nanocrystals on the irradiated area were possible protein intra-motion tracers to be tracked by DXT. Supplementary Figures S2a and S2b show AFM images of the sample surface with nAChR and gold nanocrystals. Layered structures and circular dots were identified as nAChR and gold nanocrystals, respectively. Dots with a height larger than 50 nm are coloured in red in Supplementary Figure S2c, corresponding to gold nanocrystals. We found approximately 25 gold nanocrystals in 25 μm^2^ (5 μm × 5 μm, 1 nanocrystal/μm^2^). Therefore, there are approximately 6,000 gold nanocrystals in the beam area (40 μm × 150 μm). Diffraction spots could be observed when a gold nanocrystal satisfied the conditions of Bragg's law, and we usually detected 3–5 moving diffraction spots for each measurement (100 μs/f, 100 frames). Most of them appeared at different angular positions as shown in Figure 1d and in different frames on time-resolved diffraction images, and their spots moved in different directions with different speed. Figure 1e shows the trajectories of diffraction spots from gold nanocrystals on AChBP in the presence of ACh condition. Most of the diffraction spots appeared near the expected position of Au(111) or Au(200) from incident X-ray (E = 15.2 keV in peak) and sample-to-camera distance (100 mm). Most of them came from different gold nanocrystals on the protein. We tracked each of them and analysed those motions independently. Figure 2 shows the schematic procedure of our analysis. Individual diffraction spots from gold nanocrystals can be identified as bright spots on the time-resolved diffraction images obtained by DXT measurement. Figure 2a shows three examples of diffraction spot trajectories, moving radially (coloured in green), in concentric circles (coloured in blue) and both radially and in concentric circles (coloured in red). We analysed the displacement of angles from the starting angular position in the tilting (θ) and twisting (χ) directions from the centre of the beam in the diffraction image and sample to detector distance (approximately 100 mm), as shown in Figures 2b and 2c. To compare the angular motion statistically with different experimental conditions, we drew a 2D-axes motion histogram of nAChR or AChBP at each experimental condition generated from the angular displacement of the gold nanocrystal on the protein in both the tilting and twisting directions at each time segment, e.g., 100 μs. Figure 2d shows a 2D scatterplot in normal scale obtained from the data set in Figures 2b and 2c. We can classify three types of motions based on the scatter plot distribution. The logarithm of absolute displacement within a certain time interval was used to draw a 2D histogram in this article, and the 2D histogram was normalised to obtain a probability density motion map, as shown in Figure 2e. We termed those 2D probability density maps internal motion maps.

**Figure 3:**
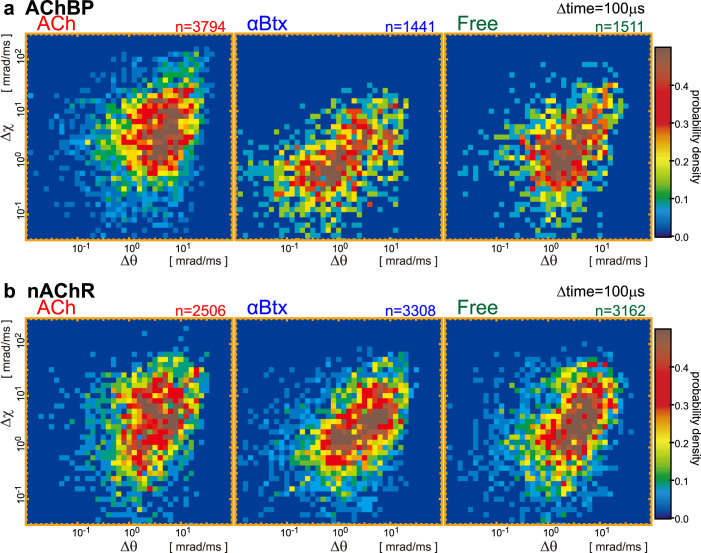
Internal motion maps of AChBP and nAChR. Internal motion probability density maps of AChBP (a) and nAChR (b) under three different experimental conditions, in the presence of either ACh or αBtx or in the absence of any agonist or antagonist. We measured the angular displacements over 100-μs time intervals in both the tilting (θ) and twisting (χ) directions, and the common logarithm of the angular velocity (mrad/ms) was used to map the probability density. The horizontal and vertical axes are the tilting and twisting directions, respectively. The number of segments (n) used to make each map is shown at the top of each map.

Figures 3a and 3b show the 2D internal motion maps of AChBP and nAChR, respectively, over a time interval of 100 μs. More than 150 diffraction spot trajectories under each DXT experimental condition were used to generate these internal motion maps. Typical open duration lifetimes obtained by patch-clamp single current recording were in the range of 1.0-10 ms for *Torpedo* nAChR^19^,^20^. We determined that continuous 100-frame measurements (total observation time of 10 ms) efficiently monitored the dynamic motions of AChBP and nAChR with 100 μs time resolution. The extent of thermal vibration in rational motion was represented as the motion map in the free condition (absence of agonist and antagonist) shown in Figure 3. It illustrates tilting and twisting motions under free conditions that ranged from 10^−1^ to 10^2^ mrad/ms. The DXT method only measures the rotational motion in the tilting and twisting directions and does not measure the translational motion. Therefore, thermal noise that related to translational motion was not reflected in the 2D-motion maps. At first analysis, because internal motion maps could not be fitted to a single 2D Gaussian function, we used cluster analysis, which is a multicomponent 2D Gaussian analysis^21^,^22^,^23^, to determine the centre of clusters in the observed 2D-axis internal motion maps. The highest, second highest, and third highest frequencies of analysed clusters are shown as a closed circle, dashed circle and cross mark, respectively, in Supplementary Figure S3. In the presence of αBtx, the tilting and twisting motions of both AChBP and nAChR were restricted, and the values were negligible compared with the motion in the presence of ACh or free conditions, as determined by cluster analysis. The magnitude of protein motion in the ligand-free condition was comparable to the protein motion observed in the presence of ACh. Next, we compared the differences of these maps between two experimental conditions at the next step.

**Figure 4:**
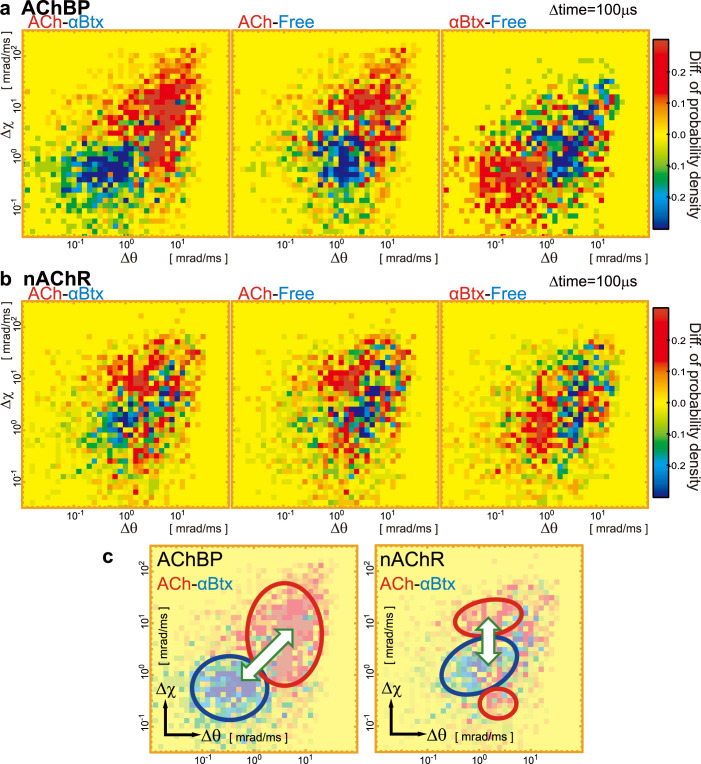
Difference internal motion maps of AChBP and nAChR between two experimental conditions. Internal motion maps were obtained for AChBP and nAChR in the presence of either ACh or αBtx or in the absence of ligands (Figures 3a and 3b). Then, maps presenting the difference in the internal motion of the protein under two different conditions (from a total of three), such as ACh-αBtx, ACh-ligand free and αBtx-ligand free, were compared. (c) Rough summary of the different internal motion maps of AChBP (left) and nAChR (right).

Figures 4a and 4b show the differences in the internal motion maps of AChBP and nAChR under three different experimental conditions. The maps were initially generated in the presence of either ACh or αBtx or in the absence of ligands. Then, maps were created that presented the difference of the internal motion of the protein under two different conditions (out of three total conditions); the ACh-αBtx, ACh-ligand free and αBtx-ligand free conditions were compared.

We found that the differences in the internal motion maps could be divided into two areas, especially in the case of the ACh-αBtx and ACh-free conditions for AChBP, as shown in Figure 4c-left. In Figure 4a, the ACh-positive area coloured in the yellow-red colour scale is distributed in the upper right, and the αBtx-free or αBtx-positive area coloured in the yellow-blue colour scale is distributed in the lower left of the map. This result means that both the tilting and twisting motions of AChBP were facilitated in the presence of ACh and inhibited in the presence of αBtx, and these tendencies were observed at all time intervals from 100 to 900 μs (Movie S1-AChBP). AChBP is a homopentamer, and the ACh binding site is located at the boundary between subunits, which consists of principal and complementary loops^24^. Of the principal loops, loop C is considered to be one of the most sensitive regions to conformational changes upon ACh binding. Loop C is located on the C-terminus of AChBP, which was labelled with a gold nanocrystal in our experiments. Therefore, enhanced nanocrystal motion, which was observed in the presence of ACh by DXT, reflects the conformational change of the ACh binding site of AChBP upon binding and unbinding of ACh. Figure 4b shows the difference in the internal motion maps of nAChR, which were more complicated. In the case of ACh-αBtx (nAChR), the map can be divided into three areas: an ACh-positive area with high χ motion (coloured in yellow-red scale), an αBtx-positive area (coloured in yellow-blue scale), and an ACh-positive area with low χ motion (coloured in yellow-red scale), as shown in Figure 4c-right. This behaviour was also observed at different time intervals ranging from 100 to 900 μs (Movie S1-nAChR). *Torpedo* nAChR is a heteropentamer with two ACh binding sites; one is located at the interface between each α subunit and either the γ or δ subunit. Additionally, prolonged exposure to ACh induces nAChR to adopt a desensitised state, indicating that nAChR can exist in multiple conformations. We hypothesise that the complex motion pattern observed by DXT could be attributed to these heterogeneous characteristics of nAChR. Although we could not discern the motion pattern of nAChR states in the current data set, ACh-induced conformational changes in the α-subunit of nAChR were clearly observed by our method.

**Figure 5:**
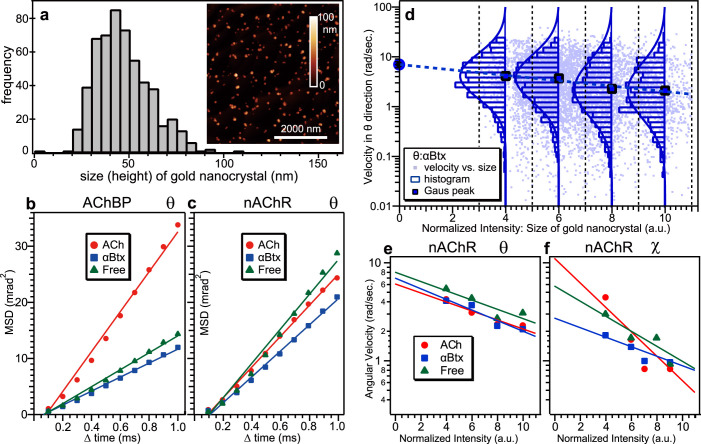
Loading test of nAChR's motion with gold nanocrystals. (a) AFM analysis of gold nanocrystals. The size (height) of gold nanocrystals ranged from 20 to 80 nm. (b–c): MSD curves for AChBP (b) and nAChR (c) in the tilting direction in the presence of ACh (red) or αBtx (blue) and in the absence of ligands (green). (d): The observed angular velocity as a function of normalised intensity of the diffraction spot. (e): The relationship between the most probable angular velocity and normalised intensity in the presence of ACh (red) or αBtx (blue) and in the absence of ligands (green).

The size of the gold nanocrystal was comparable to the size of the target proteins, and the effect of the gold nanocrystal on the protein's dynamics should therefore be considered in the DXT method. We estimated the degree of disturbance of the inherent motion of the target protein from the labelled gold nanocrystal. We determined the average size of the gold nanocrystals to be 40 nm, and the size of each gold nanocrystal bound to the target protein was between 20 and 80 nm, as shown in Figure 5a. The numbers of possible labelled positions on AChBP and nAChR were five and two, respectively. The effect of the gold nanocrystal label on the motion of the target protein was evaluated by mean square angular displacement (MSD) curves from DXT. As shown in Figures 5b and 5c, MSD curves show a simple linear relationship between the mean square angular displacement in the tilting (θ) direction and the time interval, indicating that no restrictive force was exerted on AChBP (Figure 5b) or nAChR (Figure 5c). MSD curves should be distorted when additional force is exerted on the target protein^25^. The slopes of the MSD curves obtained from AChBP and nAChR were comparable in value, indicating that the majority of gold nanocrystals did not bridge across multiple nAChRs. Figure 5d shows the relationship between nAChR's angular velocity in the θ direction and the normalised intensity of the diffraction spot in the presence of αBtx. Normalised intensity was inferred to be the size of the gold nanocrystal. Inset histograms constructed by recording the segment of angular displacement under a specified normalised intensity range illustrate the absolute angular velocity in the θ direction. All histograms could be fitted to normal Gaussian distributions, and Gaussian peaks in each normalised intensity range were plotted as blue circles. We found that larger gold nanocrystals decreased the angular velocity, and there was a simple relationship between the peak value of the angular velocity and the normalised intensity. The original motions of the nAChR in the presence of αBtx, without gold nanocrystal labelling, could be determined by extrapolation to be approximately 10 rad/sec (dashed line) in Figure 5d. The same analyses were performed in the presence of ACh and free of both agonist and antagonist (free). Figures 5e and 5f show the relationship between the Gaussian peak of angular velocity within the specified normalised intensity range and normalised intensity in the θ (Figure 5e) and χ directions (Figure 5f). The inherent angular velocities in the θ and χ directions were obtained from the intercept of the linear regression between the logarithms of angular velocity and normalised intensity (Table 1).

## Discussion

We analysed the agonist- or antagonist-induced structural dynamic of AChBP and nAChR at the single molecule level using DXT methodology. We immobilised an internal motion tracer, a gold nanocrystal, on the ligand-binding domain of nAChR and the C terminal of AChBP and found that the combined tilting and twisting motions of the proteins were enhanced by ACh binding. We determined that ACh induced tilting and twisting motions for both AChBP and nAChR, whereas αBtx restricted these motions for both proteins. We characterised the area of the map with low χ motion as a desensitised state of nAChR, shown in the right side of Figure 4c.

Averaged size of the gold nanocrystals was approximately 40 nm in diameter (Figure 5a), and its size was comparable to the size of AChBP or nAChR. The gold nanocrystal distributed in low density such as approximately 1 particles/μm^2^ (Supplementary Figure S2) in DXT sample substrate. Therefore, it is reasonable to think that one gold nanocrystal interact with one protein molecule as shown in Figures 1a and 1b, and the observed moving diffraction spots were reflected from the single molecule of AChBP or nAChR. The number of binding sites between gold nanocrystal and AChBP or nAChR may differ among proteins. Especially, AChBP forms homopentamers and has Met-tag in each domain for binding gold nanocrystal. The number of the binding sites will affect the frequency of the observed diffracted spot motions but not the mode of the motions since each ligand-induced molecular motion of the subunit is equivalent.

The size effect from the gold nanocrystal on the objective proteins should be considered in the DXT method. The molecular dynamics simulation for peptide folding with gold nanocrystal in DXT showed that the nanocrystal did not affect the global-minimum state but did affect the protein folding pathway^26^. The size of the gold nanocrystal was comparable to the size of AChBP or nAChR (Figure 5a); therefore, the crystal's effect was smaller than in the case of peptide folding. We found that the original motions in the tilting and twisting directions were affected by the size of the gold nanocrystal (Figure 5d) and could be determined by the intercept of the relationship between the most probable angular velocity and normalised intensity (Figures 5e and 5f). The nAChR intercept value of the twisting (χ) direction in the presence of ACh was high (11.1 rad/sec) compared with the condition that was free of agonist or antagonist (5.8 rad/sec) and in the presence of αBtx (2.7 rad/sec); however, the intercept value of the tilting (θ) direction was almost identical in all three experimental conditions (Table 1). As the gold nanocrystal was immobilised on the ligand-binding domain of nAChR, the majority of the ligand-induced motion was twisting. Channel opening was expected to involve a tilting motion of the transmembrane channel domain, and such tilting motions should be detected if a gold nanocrystal is specifically immobilised on the transmembrane domain of nAChR. Regarding the value of rotation, we can convert the observed angle of rotation into translational length by assuming a centre axis for rotation (*d = rα*; where *d* is the translational length, *r* is the radius of rotation, and *α* is the angle of rotation in radians) as described in Supplementary Figure S4. The intercept values of rotation in the presence of ACh and αBtx are 11.1 mrad/msec and 2.7 mrad/msec, respectively. These values are 0.044 nm/msec (ACh) and 0.011 nm/msec (αBtx) in translational length if the centre of rotation is the centre of nAChR (r = 4 nm), which demonstrates the ability of DXT to distinguish motion at a picometre scale. Because our assumed radius of rotation for estimating the translational length could be larger than the real rotation, the translational length could be overestimated. A recent electron crystallography study demonstrated that ACh binding to the α subunit of nAChR triggered a distortion in the ligand-binding domain, which involved the outward displacement of the extracellular portion of the β subunit by approximately 1 Å^14^. A normal mode analysis, based on structural information on snail AChBP and *Torpedo marmorata* nAChRs, showed that the opening of the α7-nAChR channel pore was caused by a twisting motion of the protein: specifically, the opposing rotation of the extracellular and trans-membrane domains^27^. These findings are consistent with our result that ACh enhances the motion of the ligand-binding domains of AChBP and nAChR.

In regard to radiation damage from the incident X-ray, ligand-induced motion was detected by the DXT method within 10 ms of measurement time; therefore, biological activity, at least binding activity to ACh or αBtx, remains in our experimental condition. The X-ray dose in our DXT measurement was approximately 4800 Gy. The tolerable x-ray dose depends on the protein and experimental conditions and has often been evaluated for protein crystals. For example, a lysozyme crystal can endure 500 kGy at room temperature^28^.

The concentrations of ACh and αBtx were fixed at 100 μM and 50 nM, respectively, in these experiments. In principle, we can estimate the concentration of the agonist or antagonist based on the frequency of the specific motion associated with the agonist or antagonist. We found that the tilting and twisting motions were enhanced by acetylcholine and degraded by αBtx in the experimental buffer. However, the degree of motion also depended on the size of gold nanocrystal and how the gold nanocrystal attached to the target protein. Therefore, there are several uncertain factors in distinguishing specific motion based on the degree of rotational motion by DXT. We could estimate the concentration of agonist or antagonist if such uncertain factors are eliminated in the future.

In addition to the DXT method, single-molecule fluorescence resonance energy transfer (single-molecule FRET) method^29^,^30^ is a candidate for investigating the intra-molecular dynamics of protein machinery at the single molecule level. FRET relies on the distance-dependent transfer of energy from donor fluorophores to acceptor fluorophores and is one of the novel tools available for measuring nanometre-scale distances and changes in distances between the two fluorophores. However, it is difficult to measure the rotational intramolecular structural changes of a single protein molecule using FRET because it cannot directly detect rotational intramolecular structural changes; that is, single-molecule FRET cannot detect rotational motion that does not change the distance between the donor and the acceptor. The persistence of the FRET method mostly depends on the stability of the fluorophores, which limits the experimental conditions, such as pH and temperature, and FRET suffers from fluorophore bleaching. The DXT method can be executed under any experimental condition that the labelled nanocrystal can withstand, and detects the rotational motion of a nanocrystal immobilised on the target protein with high precision, such as on a milliradian scale.

In summary, dynamic 3D single molecule observations of AChBP and nAChR, with picometre accuracy and 100 μs time resolution, were achieved using DXT. When ACh binds to AChBP and nAChR, microsecond combinatorial molecular dynamics occur in both the tilting and twisting directions. Thus, DXT is highly precise and can detect important intramolecular motions in all channel proteins. Our analyses revealed that a channel protein's biaxial angular motion maps are useful for understanding the function of the channel in real time and on an atomic scale.

## Methods

### Proteins

AChBP cDNAs, originally isolated from the *Aplysia kurodai* CNS (S. Kobayashi, B.-K. Kaang and T. Kubo, manuscript in preparation) was recombinantly expressed in *E. coli* and purified (see Supplementary Methods). A His-tag was inserted at the N-terminal of AChBP to immobilise the protein to the substrate surface via Ni-NTA, and a Met-tag was inserted at the C-terminus of AChBP to immobilise the gold nanocrystal. A *Torpedo californica* electric organ was used to prepare nAChR-rich membrane vesicles, which permeate cations in an agonist-dependent manner^31^, as described by Brisson and Unwin^32^, with minor modifications. The details of the preparation are described in the Supplementary Methods. The F(ab')2 fragment antibody against α-subunits of nAChR is also described in Supplementary Methods.

### Sample Preparation for Diffracted X-ray Tracking

A 50-μm-thick polyimide film (Du Pont-Toray, Tokyo, Japan) was coated with chromium (10 nm) and gold (25 nm) by vapour deposition and used as the substrate surface. The signal to noise ratio of the diffraction spot from the gold nanocrystal was high enough to be distinguished from the powder diffraction of a 25-nm-thick gold substrate. AChBP was immobilised on the substrate surface through His-tag chemistry. First, gold substrates were reacted with 2 mM of 3,3′-dithiobis[N-(5-amino-5-carboxypentyl)propionamide-N′,N′-diacetic acid] dihydrochloride (Dithiobis C2-NTA, Dojindo, Japan) in ethanol overnight at 4°C. Then, NTA functionalised substrates were washed with ethanol and Milli-Q water and soaked in a 100 mM NiSO_4_ solution (in 50 mM MOPS, pH = 7.0) for 4 hr at RT. The Ni-treated substrate was rinsed with Milli-Q water and reacted with a His-tagged AChBP solution (0.2 mg/ml in PBS) overnight at 4°C. The nAChR samples were prepared using a bifunctional chemical crosslinker to attach the protein to the gold substrate. An aliquot of succinimidyl 6-[3(2-pyridyldithio) propionamide] decanoate (LC-SPDP, Thermo Scientific) in ethanol solution was reacted with the gold-coated polyimide films overnight at 4°C. The LC-SPDP functionalised substrate was washed with ethanol and PBS buffer, and aliquots of nAChR-rich membrane vesicles were reacted with the substrate overnight at 4°C. The nAChR-substrate was washed with PBS and reacted with gold nanocrystals modified with anti α-subunit of nAChR antibody (F(ab')2) for 4 hr. The solution of gold nanocrystals reacted with AChBP-modified surface for 4 hr. The gold nanocrystal-modified surface was rinsed with PBS buffer and stored in the same buffer (the absence of 100 μM ACh and 200 nM αBtx) until use. An experimental chamber was developed with a sample substrate film and a 50 μm thick spacer of polyimide film. A schematic diagram of the sample holder is shown in Supplementary Figure S5.

### Diffracted X-ray Tracking (DXT)

The intra-molecular dynamics of AChBP and nAChR were monitored by recording the Laue spot trajectories of the gold nanocrystal on the objective protein in the presence of ACh (100 μM, Nacalai, Japan), in the presence of αBtx (200 nM, Tocris bioscience), and in the absence of any ligand. X-rays from the beamline (BL40XU, SPring-8, Japan) with energy widths ranging from 14.0–16.5 keV were used for DXT measurements (Supplementary Figure S1). The diffraction spot from a gold nanocrystal can be detected when the lattice planes of the gold nanocrystal satisfy the Bragg's law (2*d* sin*θ* = *n λ*, where *d* is the inter-planar spacing, *θ* is the angle between the incident beam and the relevant crystal planes, *n* is an integer, and *λ* is the wavelength of the incident beam), and therefore the energy width of the incident beam determines the dynamic range of the angular position of the gold nanocrystal in tilting (*θ*) direction. The size of the X-ray beam at the sample position was 40 μm (vertical) by 150 μm (horizontal) (full width at half maximum). The diffraction spots from each nanocrystal were monitored using an X-ray image intensifier (150 mm in diameter, V5445P, Hamamatsu photonics, Japan) and a CMOS camera (1024 pixel × 1024 pixel, SA 1.1, Photoron, Japan) at room temperature (25°C), and temperature was not controlled during the experiment. There is no critical temperature increase due to the incident X-rays because the functional motion of the protein, bacteriorhodopsin and cheperonin could be measured without cooling devices in previous DXT experiment^7^,^33^,^34^. The specimen-to-camera distance was approximately 100 mm, and it was calibrated by diffraction from the gold film. Angular resolution of the measurement is described in Supplementary Methods.

Gold nanocrystals were fabricated by epitaxial growth^35^ on either a NaCl (100) or KCl (100) substrate (7 mm × 7 mm area) under 10^−4^ Pa vacuum condition. AFM images of the gold nanocrystal on the KCl surface (Figure 5a) showed 1,000 particles in 100 μm^2^ (10 μm × 10 μm). They were detached by dissolving with detergent (n-decyl-β-D-maltoside, 50 mM MOPS, pH = 7.0; Dojindo, Japan) for AChBP or by dissolving in MOPS buffer with F(ab')2 fragment antibody against α-subunit nAChR (0.1 mg/ml). The gold nanocrystal for nAChR was modified with the F(ab')2 fragment through thiol-Au chemistry. The final concentration of gold nanocrystal in dissolved solution was approximately 2 × 10^9^ particles/ml. An aliquot of gold nanocrystal solution (50 μl) was reacted with the protein-modified surface (7 mm × 7 mm area). The size of the nanocrystals was estimated by the height of the particle observed by tapping mode AFM (MultiMode NanoScopeIIIa, Veeco, CA) in air (Figure 5a).We detected only 3–5 moving diffraction spots in average for each measurement, the detection probability was low considering the density of gold nanocrystal (about 1 nanocrystal/μm^2^) on sample surface (Supplementary Figure S2) and the beam size of incident X-ray (40 μm × 150 μm). The possible reason of such low detection probability is described in Supplementary Methods.

Custom software written for IGOR Pro (Wavemetrics, Lake Oswego, OR) was used to analyse the diffraction spot tracks and trajectories. The time series of the angular position of the gold nanocrystal on the target protein in the tilting and twisting directions were smoothed with a 3-point moving average filter to reduce high-frequency noise. The intensity of the diffraction spot from gold nanocrystal varies depending on the tilting angular position of diffraction spot and the corresponding flux of incident X-ray, therefore the intensity of the diffraction spot was normalised by considering the incident X-ray beam spectra (Supplementary Figure S1). We examined the trajectories from diffraction spot from Au (111) to analyse relationship between the angular velocity and normalised intensity in Figure 5d. To analyse the motion statistically, we drew a 2D-Axes motion histogram of nAChR or AChBP. The value of angular motion in the tilting and twisting direction at fixed time interval were used to make the 2D-histogram, which was normalised to obtain probability density.

The X-ray dose (*D*) was estimated from the following equation: *D* = *(μ/ρ) n t e E/A*, where *μ/ρ* is the mass absorption coefficient of water, *n* is the photons per second (10^13^), *t* is the radiation time (10 ms), *e* is the electron charge, *E* is the photon energy (E = 15.2 keV), and *A* is the radiation area (50 μm × 120 μm).

## Author Contributions

H.S., Y.Su. and Y.C.S. performed and analysed DXT experiments. Y.N. and A.M. prepared nAChR sample. S.K., Y.Sh., W.C. and T.K. prepared mutant AChBP sample. K.N. and M.O. analysed DXT results with cluster analysis. K.I., N.O., N.Y. and Y.C.S. arranged instruments for DXT experiments at SPring-8. T.K., A.M. and Y.C.S. conceived and designed the experiments. H.S. and Y.C.S. wrote manuscript.

## Supplementary Material

Supplementary Information Supplementary Information

Supplementary Information Supplementary Video 1

## Figures and Tables

**Table 1:** Inherent angular velocity of nAChR in the θ and χ directions. Those values were obtained from the intercept of the linear regression between the logarithm of angular velocity and normalised intensity (Figures 5e and 5f)

condition		Intercept θ (rad/sec.)	Intercept χ (rad/sec.)
nAChR	ACh	10^0.785^	10^1.05^
		6.1	11.1
	αBtx	10^0.844^	10^0.436^
		7.0	2.7
	Free	10^0.904^	10^0.764^
		8.0	5.8
