# Supplementary material for: Real Time Ligand-Induced Motion Mappings of AChBP and nAChR Using X-ray Single Molecule Tracking
Source: Sci Rep. 2014 Sep 16;4:6384. doi: 10.1038/srep06384 (PMC4165275; doi:10.1038/srep06384)
Supplement: Supplementary Information [file srep06384-s1.pdf]

Supplementary information

Real Time Ligand-Induced Motion Mappings of AChBP and nAChR Using X-ray Single Molecule Tracking

Hiroshi Sekiguchi[1,2], Yasuhito Suzuki[1,3], Yuri Nishino[4,5], Suzuko Kobayashi[6], Yoshiko Shimoyama[6], Weiyan Cai[6], Kenji Nagata[3], Masato Okada[3], Kouhei Ichiyanagi[1,3], Noboru Ohta[2], Naoto Yagi[1,2], Atsuo Miyazawa[4,5,*], Tai Kubo[1,6,7*], and Yuji C. Sasaki[1,2,3,*]

1 CREST Sasaki Team, Japan Science and Technology Agency, The University of Tokyo, #609 Kiban Bldg., 5-1-5 Kashiwanoha, Kashiwa City, Chiba, 277-8561, Japan
2 Research & Utilization Division, Japan Synchrotron Radiation Research Institute, SPring-8 1-1-1 Kouto, Sayo-cho, Sayo-gun, Hyogo 679-5198, Japan
3 Graduate School of Frontier Sciences, The University of Tokyo, Kiban Bldg., 5-1-5 Kashiwanoha, Kashiwa City, Chiba, 277-8561, Japan
4 Graduate School of Life Sciences, University of Hyogo, 3-2-1 Kouto, Kamigori-cho, Ako-gun, Hyogo, 679-1297, Japan
5 RIKEN SPring-8 Center, 1-1-1 Kouto, Sayo-cho, Sayo-gun, Hyogo, 679-5148, Japan
6 Biomedical Research Institute, National Institute of Advanced Industrial Science and Technology (AIST), 1-1-1 Higashi, Tsukuba, Ibaraki 305-8566, Japan
7 Molecular Profiling Research Center for Drug Discovery, National Institute of Advanced Industrial Science and Technology (AIST), 2-4-7 Aomi, Koto-ku, Tokyo 135-0064, Japan

Supplementary Methods

Preparation of recombinant AChBP

AChBP cDNA, originally isolated from the *Aplysia kurodai* CNS (S. Kobayashi, B.-K. Kaang and T. Kubo, manuscript in preparation), was amplified by PCR using a forward primer encoding a hexahistidine- (His-) tag sequence and a reverse primer encoding a Met-tag (MGGMGGM, Okada et al. Bioconjugate Chemistry 22:887 (2011)) sequence. The amplified DNA fragment was subcloned into the plasmid pQE-30 (QIAGEN). Met-tagged AChBP was expressed in *E. coli* XL1Blue MRF (Stratagene, La Jolla, CA) and induced by 1 mM isopropyl β-D-1-thiogalactopyranoside (IPTG). The AChBP accumulated in inclusion body pellets was solubilised in solubilisation buffer (50 mM CHAPS pH 11, 0.3% N-lauroylsarcosine and 1 mM DTT). Solubilised AChBP was refolded using a Protein Refolding Kit (Merck, Germany) and purified with Ni-NTA Agarose (QIAGEN), according to the manufacturer's protocol. To check the quality of the refolded and purified AChBP, it was assessed for binding of several nAChR ligands by Biacore (GE Healthcare, NJ). The dissociation constant of AChBP was 29 nM for αBtx, which is comparable to the dissociation constant of nAChR. The immobilisation efficiency of gold nanocrystal for AChBP was enhanced by inserting three Met residues at the C-terminal of AChBP.

Preparation of nAChR

A *Torpedo californica* electric organ was used to prepare nAChR-rich membrane vesicles, which permeate cations in an agonist-dependent manner (H.-P. P. Moore et al., Proc. Natl. Acad. Sci. U. S. A. 77, 4509–13 (1980), as described by Brisson and Unwin (J. Cell Biol. 99:1202 (1984).), with minor modifications as follows: 40 g of electric organ was homogenised using an Omni Mixer Homogeniser (OMNI International) for 1 min at maximum speed in 50 ml of buffer A (400 mM NaCl, 10 mM N-ethylmaleimide, 20 mM sodium phosphate, pH 7.4) supplemented with 1/2 concentration of Complete Protease Inhibitor Cocktail (Roche). After centrifugation at 6,000 rpm for 10 min at 4°C (Beckman JLA-10.500), the supernatant was filtered through four layers of sterile gauze. The filtrate was centrifuged at 20,000 rpm at 4°C for 30 min in a P45AT rotor (Hitachi). The pellet was resuspended in 20 ml of buffer A using a Potter-Elvehjem homogeniser and centrifuged at 30,000 rpm at 4°C for 50 min using a P45AT rotor. The pellet was resuspended in 50 ml of buffer B (100 mM sodium phosphate, pH 7.0) using a Potter-Elvehjem homogeniser supplemented with 1/2 concentration of Complete Protease Inhibitor Cocktail and then sealed in a glass cylinder tube at 4°C for 7 days to spontaneously form a density gradient. The suspension was divided into 2-ml aliquots, using a disposable pipette tip and taking care not to disturb the remaining portion. The collected fractions were examined by transmission electron microscopy, and fractions containing nAChR-rich membrane vesicles were used for analysis.

Preparation of F(ab)2 fragment antibody against nAChR

The F(ab')2 fragment antibody against nAChR was prepared as follows. Hybridoma cells producing anti-nAChR IgG1 monoclonal antibody, mAb 35, were cultured at 37°C with 7.5%

CO_2 in Iscove's DMEM (Thermo Scientific) containing 20% foetal bovine serum and 50 μg/ml gentamicin. The culture medium was gradually exchanged to HB basal medium (Irvine Scientific) supplemented with HB101 Lyophilised Supplement (Irvine Scientific). The hybridoma cell culture supernatant was loaded onto a 1 ml Protein G Sepharose 4 Fast Flow column (GE Healthcare). IgG1 was eluted with 0.1 M glycine buffer at pH 2.8, and the eluate was neutralised by adding 1 M Tris-HCl at pH 9.0. The F(ab')2 fragment antibody was produced with a Pierce Mouse IgG1 Fab and F(ab')2 Preparation Kit (Thermo Scientific) according to the manufacturer's protocol. Briefly, IgG1 was incubated overnight at 37°C with ficin that was immobilised on agarose resin. The F(ab')2 fragments were separated from the Fc fragments and undigested IgG1 by size-exclusion chromatography on tandem Superdex 200 HR10/30 columns (GE Healthcare) with running buffer (100 mM NaCl, 20 mM sodium phosphate, pH 7.0) at 4°C. The number of possible positions for the F(ab')2 fragment on nAChR is two (two α–subunits of nAChR), and the F(ab')2 fragment could bind to either of the two sites, considering the reaction efficiency of F(ab')2 fragment for nAChR. A sample of nAChR formed a supported lipid bilayer on the substrate surface. Although the orientation of nAChR on the substrate surface could not be controlled, the gold nanocrystal could only access the extracellular side of the nAChR lipid bilayers.

Angular resolution of DXT used for the measurement

The time-resolved diffraction images were recorded using an X-ray image intensifier (V5445P, Hamamatsu photonics) and CMOS camera (1024 pixel x 1024 pixel, SA 1.1, Photoron). The nominal entrance field of view for the x-ray image intensifier is 150 mm in diameter; and, the effective pixel size is 0.1465 mm. The peak energy of incident X-ray was 15.2 keV and the sample-to-detector distance is 100 mm in our DXT measurement, therefore a one-pixel movement of a diffraction spot in the tilting (θ) direction corresponds to 0.7 mrad/pixel(@15.2 keV). Most of the diffraction spots from gold nanocrystal are obtained at 36.4 mm from the beam centre, considering the d-spacing of Au (111) (d=2.35 Å). The length in 36.4 mm corresponds to 248.5 pixels in our set-up, and the circle that was 248.5 pixels in radius corresponds to approximately 1560 pixels in circumference. Therefore, a one-pixel motion in the twisting (χ) direction corresponds to 4.0 mrad/pixel@15.2 keV.

Low detection probability of diffraction spot from gold nanocrystal

We detected only 3-5 moving diffraction spots from gold nanocrystal in average for each measurement (100 μs/f, 100 frames), the detection probability was low considering the density of gold nanocrystal (about 1 nanocrystal/μm^2) on sample surface (Supplementary Figure S2) and the beam size of incident X-ray (40 μm x 150 μm). We think that primary reason for such low detection probability of diffraction spots comes from the low quality of gold nanocrystal. Since large amounts of gold nanocrystals were needed for DXT measurement, we fabricated gold nanocrystals by epitaxial growth on NaCl (100) or KCl (100) surface in 10^{-4} Pa vacuum condition (not in ultra-high vacuum condition). At current our fabrication condition, the gold nanocrystals were heterogeneously arranged on the substrate in shape and in size as shown in Figure 5a. We found that normalised intensity of diffraction spots varied in broad range (from 3 to 12 as shown in Figure 5d), and there were simple relationship between tilting or twisting

angular velocity and the normalised intensity of diffraction spot, as shown in Figures 5d, 5e and 5f. Therefore we think that diffraction spots we analysed were from certain variation of gold nanocrystals in size. We might detect diffraction spot from larger portion of gold nanocrystals, such as larger 50 nm. The method to provide large amount of high quality of gold nanocrystal with smaller size is to be established in the future.

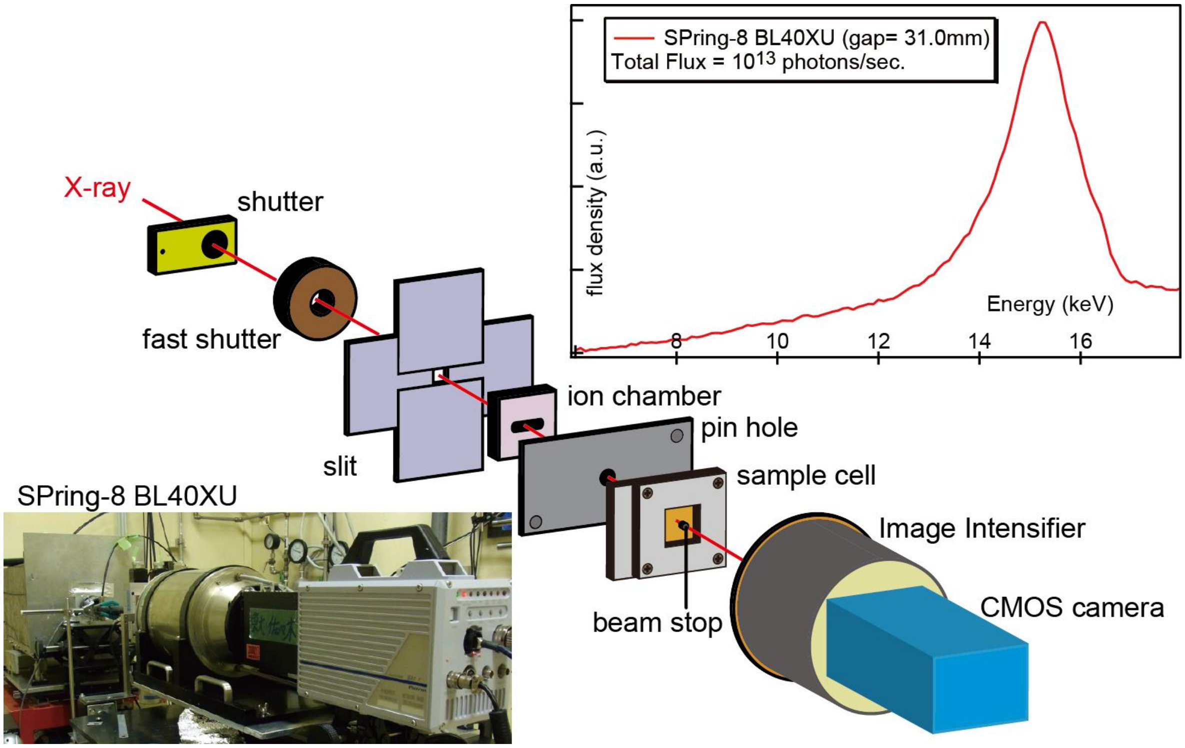

Supplementary Figure S1 Fast diffracted X-ray tracking at SPring8 BL40XU. The intra-molecular dynamics of AChBP and nAChR were monitored by recording the Laue spot trajectories of the gold nanocrystal on the objective protein. X-rays from the beamline (BL40XU, SPring-8, Japan) with energy widths ranging from 14.0-16.5 keV (undulator gap=30.1 mm) were used for DXT measurements. The photon flux density profile for the incident beam from the BL40XU in SPring-8 is shown in the inset graph (upper right, red line). The exposure time for the sample was limited to less than 15 ms to prevent X-ray radiation damage, which was achieved by combining two X-ray shutters, a solenoid shutter and a millisecond shutter (UNIBLITZ, XRS1S2PO). Impairment of protein motion was not observed under these conditions. A fast DXT measurement with 1 μs/f was achieved.

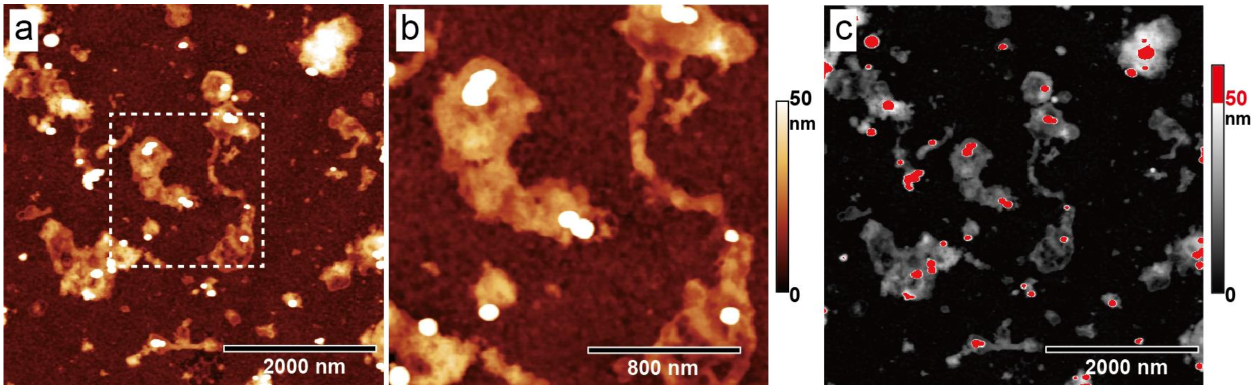

Supplementary Figure S2: AFM image of sample surface with nAChR and gold nanocrystals under dry conditions. The sample was prepared in the same way as for the DXT experiment, and the sample surface was washed with MilliQ, dried in vacuum, and imaged by MFP-3D AFM (Asylum Research, CA) equipped with a silicon tip (OMCL-AC160TS, Olympus, Japan). The images were obtained using scan sizes of 5 μm square (a) and 2 μm square (b). Layered structures and circular dots were identified as nAChR and gold nanocrystal, respectively. Dots with a height larger than 50 nm, coloured in red in (c), corresponded to gold nanocrystals. We found 25 gold nanocrystals in 25 μm^2 (5 μm x 5 μm, 1 particle/μm^2).

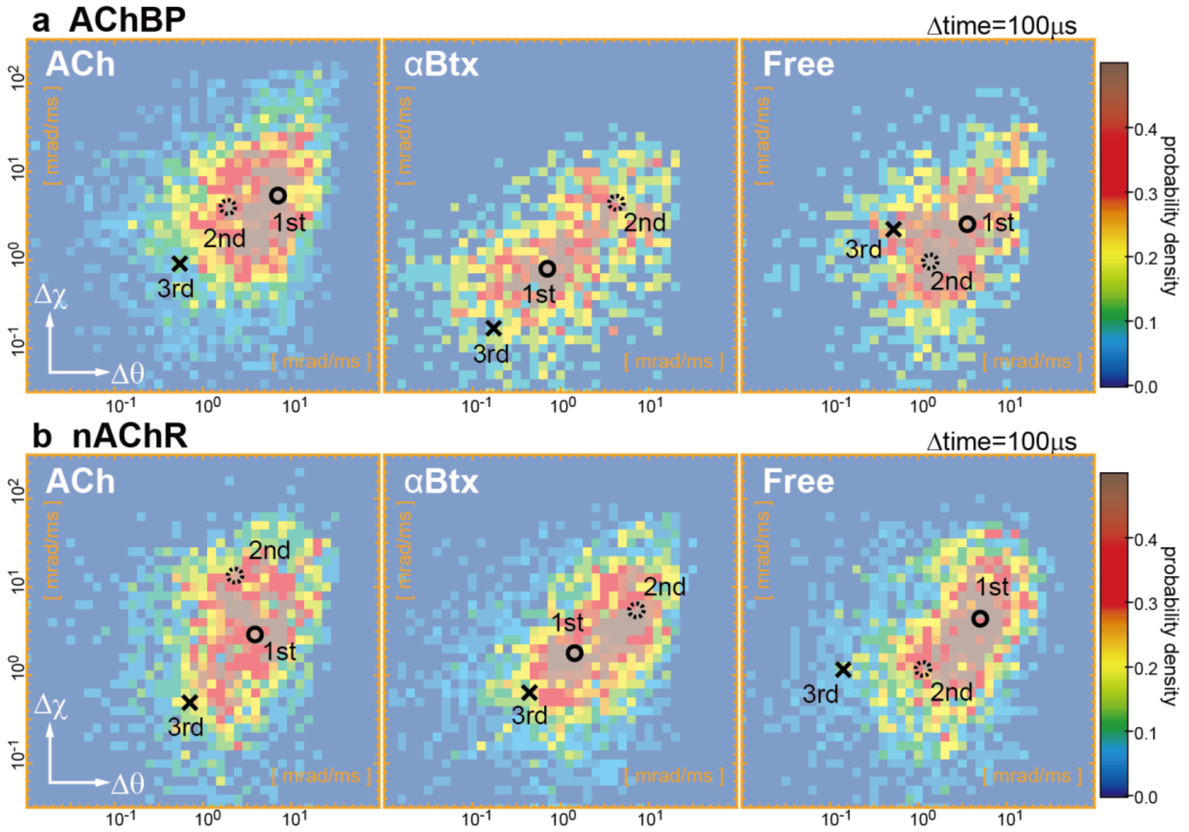

Supplementary Figure S3 Cluster analysis of 2D internal motion maps for AChBP (a) and nAChR (b). Using cluster analysis, the internal motion maps were fitted to a function composed of three 2D-Gaussian peaks. The 1st, 2nd and 3rd peaks are shown as a solid circle, dashed circle and cross mark, respectively. The fitting parameters are shown in Supplementary Table S1.

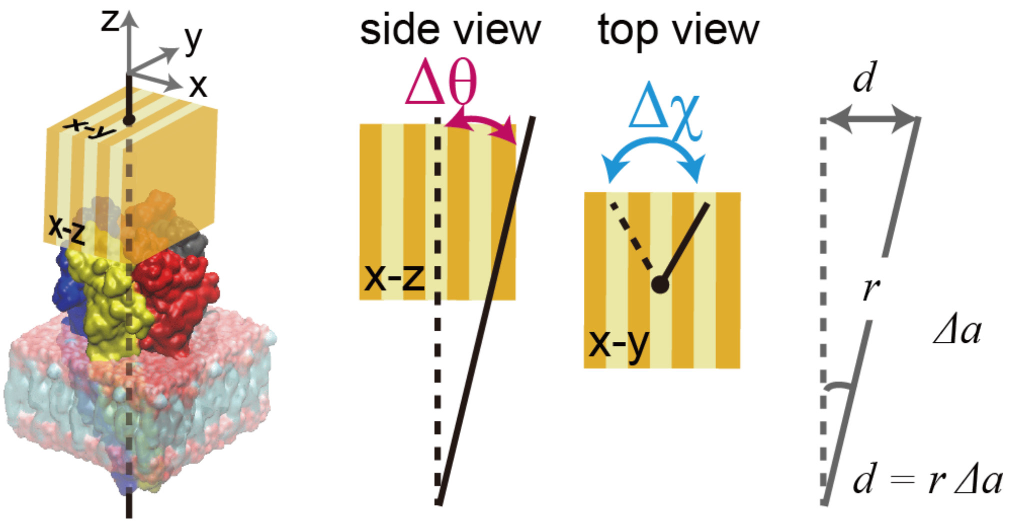

Supplementary Figure S4: Schematic of conversion from rotational motion to translational motion. By assuming a centre axis for each rotation direction, we converted the observed angle of rotation into translational length ($d = r\,\Delta a$; where d is the translational length, r is the radius of rotation, and Δa is the angle of rotation in radians).

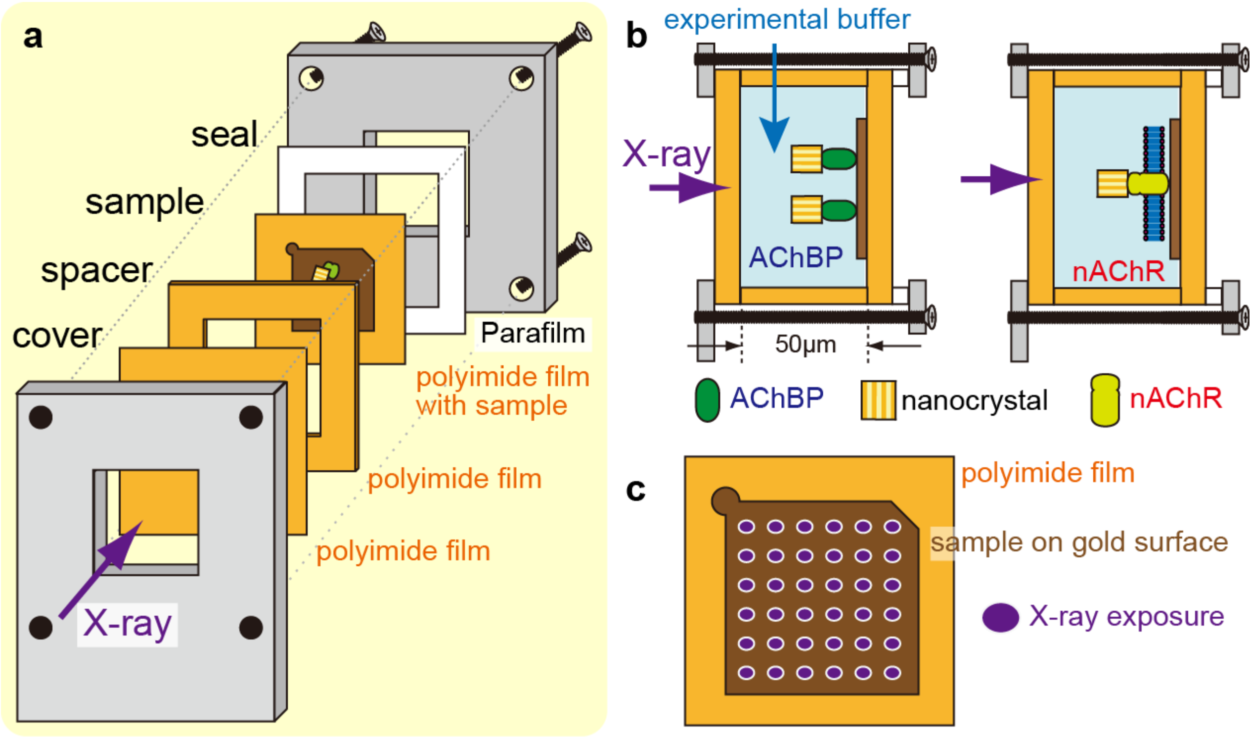

Supplementary Figure S5. Schematic drawing of DXT sample holder. (a) A sample holder was made of sample substrate film with a 50-μm-thick spacer (Kapton, Du Pont-Toray, Tokyo, Japan). The chamber, 11 mm × 11 mm × 50 μm, was covered with a 50-μm-thick polyimide film. The chamber was sandwiched by stainless steel frames and screw-clamped. (b) Side view of sample holders with AChBP (left) and nAChR (right). An AChBP molecule was immobilised on a gold-coated polyimide film using His-tag chemistry and labelled with a gold nanocrystal by a Met-tag. A sample of nAChR was immobilised on the substrate surface with chemical crosslinkers and labelled with a gold nanocrystal using an antibody that recognises the extracellular side of the nAChR α-subunit. (c) An incident X-ray irradiated one position of the sample for 15 ms, and the same measurement was then repeated, changing the irradiated area. We usually measured 36 positions (6 x 6) for one sample within a 16 mm^2 (4 mm x 4 mm) area.

		a	$\mu_{x\ [rad/s]}$	$\mu_{y\ [rad/s]}$	$\sigma_{x\ [rad/s]}$	$\sigma_{y\ [rad/s]}$	ρ
AChBP	ACh 1st	0.530	$10^{0.839}$	$10^{0.727}$	$10^{0.0855}$	$10^{0.342}$	0.393
	ACh 2nd	0.374	$10^{0.290}$	$10^{0.605}$	$10^{0.178}$	$10^{0.269}$	0.108
	ACh 3rd	0.097	$10^{-0.270}$	$10^{-0.0718}$	$10^{0.536}$	$10^{0.648}$	-0.371
	αBtx 1st	0.550	$10^{-0.164}$	$10^{-0.105}$	$10^{0.270}$	$10^{0.141}$	0.0459
	αBtx 2nd	0.310	$10^{0.627}$	$10^{0.660}$	$10^{0.157}$	$10^{0.129}$	0.0644
	αBtx 3rd	0.140	$10^{-0.766}$	$10^{-0.805}$	$10^{0.644}$	$10^{0.378}$	-0.493
	Free 1st	0.604	$10^{0.574}$	$10^{0.406}$	$10^{0.157}$	$10^{0.289}$	0.570
	Free 2nd	0.282	$10^{0.131}$	$10^{-0.0129}$	$10^{0.152}$	$10^{0.510}$	-0.183
	Free 3rd	0.115	$10^{-0.271}$	$10^{0.377}$	$10^{0.423}$	$10^{0.147}$	0.470
nAChR	ACh 1st	0.691	$10^{0.589}$	$10^{0.462}$	$10^{0.171}$	$10^{0.331}$	0.491
	ACh 2nd	0.198	$10^{0.362}$	$10^{1.12}$	$10^{0.236}$	$10^{0.0974}$	0.290
	ACh 3rd	0.111	$10^{-0.144}$	$10^{-0.305}$	$10^{0.387}$	$10^{0.644}$	-0.471
	αBtx 1st	0.477	$10^{0.172}$	$10^{0.244}$	$10^{0.128}$	$10^{0.248}$	0.222
	αBtx 2nd	0.382	$10^{0.851}$	$10^{0.744}$	$10^{0.0635}$	$10^{0.208}$	0.240
	αBtx 3rd	0.141	$10^{-0.342}$	$10^{-0.215}$	$10^{0.554}$	$10^{0.525}$	-0.188
	Free 1st	0.648	$10^{0.705}$	$10^{0.643}$	$10^{0.102}$	$10^{0.252}$	0.396
	Free 2nd	0.317	$10^{0.061}$	$10^{0.0845}$	$10^{0.226}$	$10^{0.519}$	-0.0956
	Free 3rd	0.035	$10^{-0.858}$	$10^{0.0504}$	$10^{0.637}$	$10^{0.429}$	100

Supplementary Table S1. Fitting parameters for a 2D-Gaussian function of AChBP and nAChR motions using cluster analysis. The parameters of the 1st, 2nd and 3rd peaks were determined using the following equations:

$$\mathrm{p}(\mathrm{x},\mathrm{y}) = \sum_{k=1}^{3} a_k g_k(x,y)$$

$$\mathrm{g}(\mathrm{x},\mathrm{y}) \propto \exp\left(-\frac{1}{2(1-\rho^2)}\left\{\left(\frac{x-\mu_x}{\sigma_x}\right)^2 + \left(\frac{y-\mu_y}{\sigma_y}\right)^2 - 2\rho\left(\frac{x-\mu_x}{\sigma_x}\right)\left(\frac{y-\mu_y}{\sigma_y}\right)\right\}\right)$$

Supplementary Movie S1 Different 2D-axis internal motion maps of AChBP and nAChR at various time intervals from 100 to 900 μs.
